# Early detection of glycocalyx and microvascular damage in suspected sepsis in the emergency department: the EDGE study

**DOI:** 10.1186/s13054-026-05989-9

**Published:** 2026-04-17

**Authors:** Melina Mascha Scarbeck, Marc-David Künnemann, Anna M. Hunkemöller, Carolin Christina Drost, Alexander Lukasz, Marcel Birkner, Manfred Fobker, Jerzy-Roch Nofer, Hans Vink, Hermann Pavenstädt, Philipp Kümpers, Alexandros Rovas

**Affiliations:** 1 Department of Medicine D, Division of General Internal and Emergency Medicine, Nephrology, and Rheumatology, University Hospital Münster, Albert-Schweitzer-Campus 1, 48149 Münster, Germany; 2 Clinic of Radiology, University Hospital Münster, Albert-Schweitzer-Campus 1, 48149 Münster, Germany; 3 Institute of Diagnostic and Interventional Radiology, Hannover Medical School, 30625 Hannover, Germany; 4 Central Laboratory Facility, University Hospital Münster, Albert-Schweitzer- Campus 1, 48149 Münster, Germany; 5 Institute for Laboratory Medicine, Marien-Hospital, Niels-Stensen-Kliniken, Osnabrück, Germany; 6 Glycocalyx Research Institute, Alpine, USA

**Keywords:** Endothelial glycocalyx, Capillary recruitment, Perfused boundary region, Microvascular health score, Sepsis

## Abstract

**Background:**

The prompt identification of clinical deterioration in emergency department (ED) patients presenting with infection is crucial yet challenging. Microvascular dysfunction has been linked to poor clinical outcome in critically ill patients, but it remains unclear whether its detection can predict clinical deterioration in early sepsis. This study aims to evaluate the utility of quantitative microvascular videomicroscopy for predicting clinical deterioration in patients with suspected sepsis.

**Methods:**

In this prospective observational study, 299 ED patients with suspected infection or sepsis were enrolled, with the addition of 50 healthy volunteers, 14 non-infected ED patients and 34 intensive care unit (ICU) patients with sepsis as controls. All participants underwent sublingual sidestream darkfield videomicroscopy. The GlycoCheck™ software quantified vascular density, perfused boundary region (PBR; inverse marker of endothelial glycocalyx (eGC) thickness), and the Microvascular Health Score (MVHS™), which integrated capillary density and eGC dimensions. The primary outcome was disease progression within the first week, defined as progression from infection to sepsis or increase in SOFA score in septic patients. Secondary outcomes included in-hospital and 90-day mortality, ICU admission, or a composite outcome of progression or in-hospital death.

**Results:**

Sublingual videomicroscopy revealed significant differences in all microvascular variables between ED patients, healthy volunteers and control groups, correlating with disease severity. ED patients with disease progression showed lower capillary density, higher PBR, and lower MVHS at baseline than non-progressors. In patients presenting with infection without sepsis, MVHS demonstrated strong predictive discrimination for progression (AUC 0.79, *p* < 0.0001), outperforming procalcitonin and interleukin-6. An intact microvascular phenotype (high capillary density and low PBR) markedly reduced the risk of disease progression or in-hospital mortality (OR 0.17, *p* < 0.001), whereas combined glycocalyx damage and reduced capillary density significantly increased risk (OR 2.39, *p* = 0.01).

**Conclusion:**

Quantitative sublingual videomicroscopy predicts early disease progression within the first week and stratifies patients with suspected sepsis into high and low-risk groups at ED presentation.

**Trial registration:**

Clinicaltrials.gov Identifier NCT03126032, Registration Date 20.02.2017.

**Supplementary Information:**

The online version contains supplementary material available at 10.1186/s13054-026-05989-9.

## Background

At present, no reliable method exists to predict the clinical trajectory of stable patients presenting to the emergency department (ED) with infection. In particular, current biomarkers and diagnostic technologies have shown limited efficacy in predicting the development of sepsis at an early stage, i.e., before the onset of organ dysfunction [1–4]. However, it is imperative to identify patients who are at high risk of developing sepsis or experiencing substantial clinical deterioration. Such prognostication may change clinical decision-making regarding the intensity of monitoring and therapeutic intervention. Consequently, effective sepsis forecasting in the ED may improve outcomes for patients presenting with infections.

Previous studies have emphasized the role of microvasculature in critically ill patients, indicating a potential link between microvascular dysfunction and organ failure [5]. Several indices of microvascular perfusion have been proposed to improve risk stratification and prognostication in intensive care (ICU) patients with established sepsis and septic shock [6]. Among these, the proportion of perfused small vessels (PPV) has been identified as a strong predictor of mortality in sepsis [7].

Degradation of the endothelial glycocalyx (eGC) has been associated with adverse outcomes in sepsis. The eGC, a gel-like layer of glycoproteins and proteoglycans that lines the luminal surface of the vascular endothelium, is vital for maintaining vascular homeostasis. Its disruption increases vascular permeability, promotes leukocyte adhesion and triggers microthrombosis, all of which contribute to organ dysfunction in sepsis [8–11]. Consequently, early detection of eGC breakdown may facilitate the identification of high-risk patients.

We recently developed an automated quantitative intravital videomicroscopy approach with the ability to detect even subtle changes in functional capillary density and eGC dimensions in the sublingual microvasculature [12]. In response to the Surviving Sepsis Campaign (SSC) call to improve diagnostic and prognostic tools in sepsis research [13], this study aimed to implement this novel methodology in the ED and assess its ability to predict the development of sepsis and clinical deterioration in patients presenting with clinical suspicion of sepsis.

## Methods

### Study design and population

This prospective observational study took place in the ED of the University Hospital Münster from March 2017 to November 2019 and from November 2020 to June 2022. The study was conducted in accordance with the Declaration of Helsinki, received approval from the relevant ethics committee (2016–073-f-S and amendments) and was prospectively registered (Clinicaltrials.gov: NCT03126032).

After written informed consent was obtained from the participants or their legal representatives, adult ED patients presenting with clinical suspicion of infection or sepsis (according to the Sepsis-3 definition) and requiring hospitalization (hereafter ED patients) were enrolled in a non-consecutive fashion due to logistical constraints and operator availability. As severe infection and sepsis at ED presentation often represent a clinical continuum and are managed within the same initial diagnostic and therapeutic pathway, these patients were initially analyzed as a pooled ED cohort. Additional stratified analyses according to infection or sepsis at presentation were performed to explore potential differences between these groups. Exclusion criteria were age < 18 years, pregnancy, and oral mucosal inflammation or injury affecting the sublingual microvasculature. Demographic data, routine chemistry tests, physiological variables, clinical scores (Sequential Organ Failure Assessment (SOFA) score [14] and Charlson Comorbidity Index (CCI) [15]), and sublingual videomicroscopy were recorded at baseline (Table 1). A subgroup of the first study period underwent additional sublingual videomicroscopy on day 1 (*n* = 136) and day 7 (*n* = 67) in addition to the baseline evaluation. To estimate the short-term variability of microvascular variables, a small subset of patients (*n* = 15) underwent a second measurement set approximately three hours after study inclusion.

### Control groups

Fifty apparently healthy volunteers were enrolled to establish normal ranges of the microvascular variables measured. Fourteen ED patients without infection were enrolled as disease controls. Thirty-four ICU patients with established sepsis were used as (positive) sepsis controls. All participants underwent sublingual videomicroscopy, as described in detail below. Disease controls and sepsis controls were only used for baseline comparison of microvascular variables and were not included in outcome analysis. Baseline characteristics of all groups are shown in Table 1.

### In vivo assessment of the sublingual microcirculation and glycocalyx dimensions

Microvascular measurements were performed using the GlycoCheck™ system (Microvascular Health Solutions Inc., Alpine, UT, USA), which incorporates a sidestream dark field (SDF) camera (CapiScope HVCS, KK Technology, Honiton, UK). To account for physiological variability and spatial heterogeneity of the sublingual microvasculature, two subsequent measurements were obtained and averaged, including at least a total of 6,000 vascular segments, as described in detail previously [16, 17]. All RBC-containing segments with diameters between 4 and 25 μm are automatically identified and analyzed in the video recordings. The following variables were estimated, as previously described in detail [12, 17]:

#### Glycocalyx dimensions

The GlycoCheck™ software estimates the dynamic lateral movement of the erythrocytes (RBCs) into the permeable part of the endothelial glycocalyx layer. This is expressed as the perfused boundary region (PBR_4–25 μm_; in µm), which is an inverse variable of eGC dimension. An impaired eGC allows RBCs to penetrate deeper into the endothelium, resulting in higher PBR_4–25 μm_ values [18].

#### Vascular and capillary density

Vascular density (10⁻² mm/mm²) was calculated by multiplying the number of RBC-containing vascular segments by the segment length (10 μm each). Vascular density is then normalized to the tissue surface area. Capillary density hereafter refers to the combined density of capillaries with diameters between 4 and 7 μm (Density_4−7 μm_).

#### Microvascular health score

The Microvascular Health Score (MVHS^™^) integrates capillary blood volume (CBV, calculated as capillary density * specific capillary cross-sectional area) and the PBR into a single parameter (CBV/PBR_4–25 μm_ ratio). Given that CBV declines and PBR rises during sepsis, the MVHS can be considered a composite measure of overall microvascular status. In a subgroup of patients (*n* = 134), we also assessed MVHS_dynamic_ (calculated per individual), which incorporates the concept of capillary recruitment. This is the ability to recruit additional capillaries to meet tissue metabolic demands. Previous studies have demonstrated that this autoregulatory mechanism is impaired in sepsis, leading to inadequate microvascular adaptation [12] (see Supplement for methodologic details).

### Outcome definitions

The clinical trajectory of the ED patients presenting with suspected sepsis was prospectively monitored until discharge from hospital, and mortality outcomes were assessed for all patients. The primary outcome of the study was prediction of disease progression during the initial week of hospitalization. Disease progression was defined as:

- progression from uncomplicated infection to sepsis (Sepsis-3 definition),
- any increase in the SOFA score for patients presenting with sepsis at admission. Secondary outcomes included 90-day all-cause mortality, in-hospital mortality, ICU admission and a composite outcome of either disease progression during the initial week or in-hospital death.

The outcome adjudication was performed by investigators who were blinded to the intravital microscopy measurements and not involved in the clinical management of the patients. Further exploratory sensitivity analyses were conducted in a hypothesis-generating manner to investigate additional subgroup-specific patterns and dynamic microvascular parameters.

### Statistical analysis

Data are presented as absolute numbers, percentages, and medians with interquartile ranges [IQR; 25th and 75th percentiles]. The Mann–Whitney U test, the Wilcoxon test or Kruskal-Wallis test with Dunn’s correction were used to compare continuous variables as appropriate. The chi-square test was employed for the analysis of categorical variables. Multiple testing corrections for comparisons of microcirculatory variables per diameter class were performed using the Benjamini, Krieger, and Yekutieli false discovery rate (FDR) method, with a significance threshold set at q < 0.05. Spearman’s rank correlation coefficient (rs) was used to assess correlations between variables. Univariate logistic regression was performed to identify potential predictors of disease progression. Variables with a p-value < 0.1 in the univariable analysis were included in the multivariable logistic regression model. Backward elimination was then used to refine the model, retaining only statistically significant predictors. Receiver operating characteristic (ROC) analysis was conducted to evaluate the area under the curve (AUC). Kaplan-Meier curves (log-rank testing) were employed to visualize the distribution of time-to-death variables. For 90-day mortality, patients alive at 90 days were administratively censored at day 90, and those lost to follow-up were censored at the date of last contact. Vital status at 90 days was confirmed by structured telephone follow-up and review of hospital records. For in-hospital mortality, discharge alive was treated as a censoring event. The short-term variability was assessed by coefficient of variation (CoV), which was calculated by dividing the standard deviation by the mean of the paired measurements. Contingency tables were used to calculate sensitivity, specificity, negative and positive predictive values (NPV, PPV). All statistical tests were two-sided, and a p-value of < 0.05 was considered statistically significant. Statistical analyses and figure preparations were performed using SPSS version 29 (IBM Corporation, Armonk, NY, USA) and GraphPad Prism version 10.6.1 (GraphPad Prism Software Inc., San Diego, CA, USA).

## Results

### Baseline characteristics

A total of 299 adult patients presenting to the ED with suspected sepsis were enrolled. Further clinical work-up revealed an acute infection in *n* = 174 (58.2%) and sepsis (Sepsis-3) in *n* = 125 (41.8%) (Table 1). In both groups, most patients were male (63.8% vs. 69.6%), overweight (BMI 25.2 [22.5–29.1] vs. 25.8 [22–28.7] kg/m^2^), and had a low comorbidity burden (CCI score: 0 [0–2] vs. 1 [0–4] points). The most prevalent infection focus was the urinary tract (26.4% vs. 29.6%) followed by the respiratory system (24.7% vs. 23.2%). At the time of ED presentation, the vast majority of the patients was hemodynamically stable (MAP: 93.5 [84.17–102.3] vs. 83.67 [70.67–98.5] mmHg) with low qSOFA (0 [0–1] vs. 0 [0–1] points) and SOFA scores (1 [0–2] vs. 3 [2–5] points). The pooled characteristics of the ED cohort are shown in Supp. Table 1.

Short-term variability showed a median CoV of 5.26% for PBR_4−25 μm_ and 14.98% for capillary density. Correlations of clinical and laboratory data with microvascular variables, as well as additional analyses examining temporal trajectories are shown in Supp. Figure 1–3.

**Table 1:** Baseline characteristics of all groups

Variable	Healthy controls	Disease controls	ED infection patients	ED sepsis patients	ICU sepsis patients
Number of participants (n; %)	50	14	174	125	34
Female sex (n; %)	29 (58)	7 (50)	63 (36.2)	38 (30.4)	8 (24)
Age (years, median (IQR))	54 (33–60)	58 (44–74)	63 (44–76)	67 (56–79)	66 (56–78)
BMI (kg/m^2^, median (IQR))	24.3 (22.7–27.2)	26.6 (24–28.7)	25.2 (22.5–29.1)	25.8 (22–28.7)	25.8 (21.93–29.2)
CCI score (median (IQR))	-	0	0 (0–2)	1 (0–4)	2 (1–3)
qSOFA score (points, median (IQR))	-	0 (0–0)	0 (0–1)	0 (0–1)	-
SOFA score (points, median (IQR))	-	0 (0–1)	1 (0–2)	3 (2–5)	10 (8–13)
Sepsis on admission (n; %)	-	-	0 (0)	125 (100)	34 (100)
**Focus of infection** (n; %)					
Urinary tract	-	-	46 (26.4)	37 (29.6)	-
Respiratory tract	-	-	43 (24.7)	29 (23.2)	-
Gastrointestinal tract	-	-	44 (25.3)	19 (15.2)	-
Skin/soft tissue	-	-	11 (6.3)	12 (9.6)	-
Other (unknown, CNS, cardiac, etc.)	-	-	30 (17.2)	28 (22.4)	-
**Outcomes**					
Disease progression (n; %)	-	-	17 (9.8)	36 (28.8)	-
90-day mortality (n; %)	-	-	6 (3.5)	16 (12.8)	-
In-hospital mortality (n; %)	-	1 (7.1)	5 (2.9)	13 (10.4)	13 (38.2)
ICU admission (n; %)	-	-	10 (5.77)	29 (23.2)	34 (100)
Composite outcome (n; %)^+^	-	-	20 (11.5)	38 (30.4)	-
**Laboratory data (median (IQR))**					
Creatinine (mg/dl)	0.8 (0.7–1.7)	0.9 (0.8–1.1)	1 (0.8–1.4)	1.6 (1.03–2.48)	2.15 (1.28–3.23)
CRP (mg/dl)	0.5	0.5 (0.5–0.6)	9.3 (3.6–17.6)	11.1 (5.3–19.75)	22.4 (16.9–33.3)
IL-6 (pg/ml)	2	2 (2–5.3)	57 (27–155)	130 (53–520)	452 (137–1121)
PCT (ng/ml)	0.02 (0.02–0.05)	0.02 (0.02–0.06)	0.27 (0.12–0.8)	1.43 (0.41–7.70)	17.43 (2.04–52.44)
Lactate (mmol/l)	1.1 (0.9–1.4)	1.5 (0.98–2.03)	1.2 (0.9–1.6)	1.4 (1–2.1)	1.7 (1.08–2.08)
**Macrocirculation data (median (IQR))**					
MAP (mmHg)	-	101.5 (95.58–110.67)	93.5 (84.17–102.3)	83.67 (70.67–98.5)	71.83 (66.5–81.08)
Heart Rate (pulse/min)	-	84 (65–98)	90 (77–101)	85 (76–102)	90 (75–102)
Respiratory Rate (breaths/min)	-	16 (14–16)	16 (14–18)	16 (15–20)	21 (18–26)
Temperature (°C)	-	36.75 (36.4–37)	37.6 (36.8–38.5)	37.5 (36.65–38.5)	36.9 (36.38–37.7)
**Microvascular data (median (IQR))**					
Density_4−7 μm_ (10^− 2^ mm/mm^2^)	95.4 (68.03–119.1)	85.99 (63.30–143.80)	80.19 (60.32–113)	67.37 (50.83–86.54)	59.29 (40.85–97.88)
PBR_4−25 μm_ (µm)	2.12 (1.93–2.24)	2.05 (1.98–2.14)	2.32 (2.16–2.48)	2.38 (2.24–2.51)	2.48 (2.33–2.63)
MVHS (points)	3.34 (2.52–4.31)	3.26 (1.91–5.04)	2.31 (1.53–3.46)	1.98 (1.17–2.6)	1.53 (1.2–2.70)

^+^composite outcome: disease progression or in-hospital mortality Abbreviations: BMI = body mass index, CCI score = Charlson Comorbidity Index, CRP = C-reactive protein, ED = emergency department, IL-6 = interleukin-6, IQR = interquartile range, MAP = mean arterial pressure, MVHS = microvascular health score, PBR = perfused boundary region, PCT = procalcitonin, qSOFA score = quick SOFA score, SOFA score = Sequential Organ Failure Assessment score

**Fig. 1:**
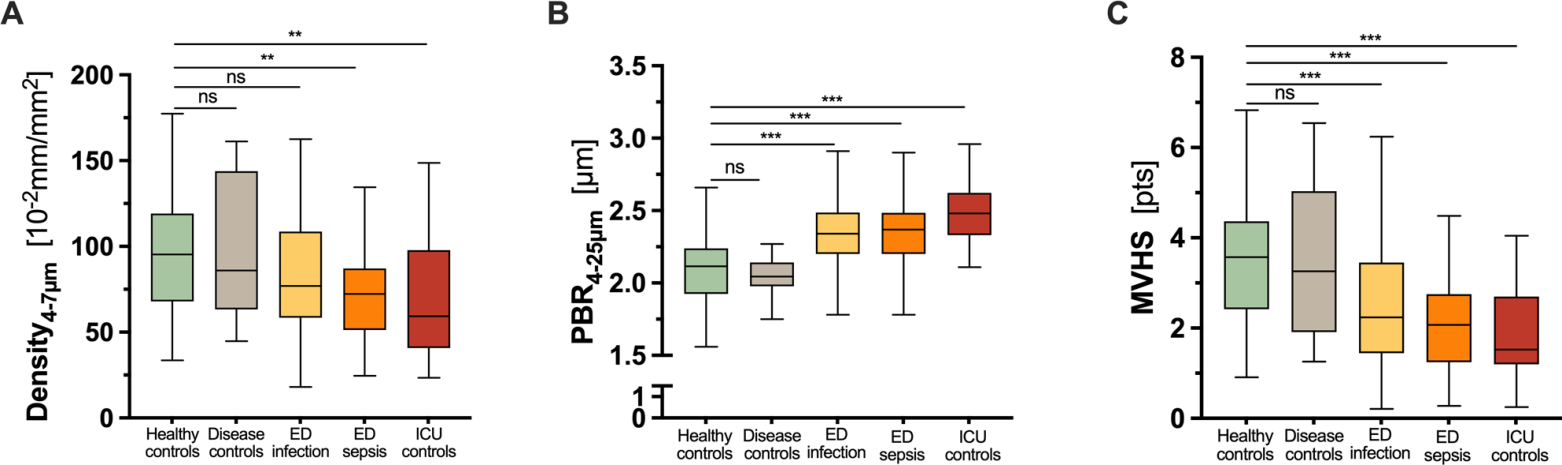
Characterization of microvascular damage in ED patients compared to controls. Boxplots showing (**A**) capillary density (Density_4−7 μm_), (**B**) perfused boundary region (PBR_4−25 μm_), and (**C**) Microvascular Health score (MVHS) in healthy controls (green; n = 50), non-infected ED patients (disease controls, grey; n = 14), ED patients with infection (yellow; n = 174) or ED patients with sepsis (orange; n = 125), and ICU controls with sepsis (red; n = 34). Groups represent a clinical severity spectrum ranging from healthy controls and non-infected ED patients to ED patients with infection or sepsis and ICU patients with established sepsis. *p < 0.05, **p < 0.01, ***p < 0.001, ns: not significant

Despite the overall low disease severity, ED patients with infection or sepsis showed reduced capillary density and higher PBR values on admission compared to healthy volunteers and disease controls (Fig. 1A, B). Notably, there was only marginal additional worsening of PBR and capillary density in the ICU controls compared to ED patients, despite considerably higher SOFA scores (ED infection: 1 [0–2] vs. ED sepsis: 3 [2–5] vs. ICU sepsis: 10 [8–13] points). As a composite metric, the MVHS exhibited a consistent decline across the various groups (Fig. 1 C). These findings indicate that the onset of microvascular changes in patients with acute infection occurs early and is detectable by our methodology already at the time of presentation in the ED.

### Primary outcome

#### Disease progression in the ED cohort

Among the 299 ED patients, 53 (17.7%) experienced disease progression during the initial week of hospitalization. The highest SOFA score during the first week (SOFA_max_) in progressors was 5 [3–9] points. ED patients who progressed were older (70 [57–80.5] vs. 63 [48–76] years, p = 0.04), had more comorbidities (CCI: 1 [0–4] vs. 0 [0–2] points, p < 0.001), and showed worse laboratory results and a slightly higher SOFA score (3 [2–5] vs. 2 [1–3] points, p < 0.001) at baseline, compared to non-progressors (Supp. Table 1). A detailed per-diameter analysis of vascular density revealed a distinct reduction in capillaries with a diameter of less than 8 μm in the ED subgroup with disease progression (D4: −30.6% (q < 0.01), D5: −41.1% (q < 0.001), D6: −35.9% (q < 0.0001), and D7: −21.8% (q < 0.001) when compared to healthy controls. Conversely, no such reduction was seen in non-progressors. Accordingly, the capillary density (Density_4−7 μm_) was significantly lower in ED patients who experienced progression compared to those who did not (62.2 [43.01–74.55] vs. 77.9 [59.52–105] 10^− 2^ mm/mm^2^, *p* < 0.001). Progressors also exhibited significantly higher PBR_4−25 μm_ values (2.39 [2.26–2.58] vs. 2.34 [2.18–2.48] µm, *p* = 0.03) and lower MVHS scores (1.52 [0.99–2.19] vs. 2.24 [1.53–3.38] pts, *p* < 0.001) at baseline (Supp. Figure 4).

Univariable regression analysis of the 299 ED patients revealed that both Density_4−7 μm_ and PBR_4−25 μm_ – or alternatively the composite MVHS – were associated with disease progression. In the multivariable model, heart rate, CCI and PCT remained significant predictors, while microvascular variables retained independent associations with disease progression, indicating that microvascular variables provide prognostic information beyond comorbidities, macrocirculatory parameters and traditional markers of inflammation (Table 2).

**Table 2:** Univariable and multivariable logistic regression with disease progression in the ED cohort (*n* = 299) as the dependent variable

Independent variables	Univariable		Multivariable	
	OR (95% CI)	*p*-value	OR (95% CI)	*p*-value
Sex (female/male)	0.66 (0.34–1.28)	0.21	-	-
Age (years)	1.02 (0.99–1.03)	**0.06**	1.02 (1–1.04)	0.12
CCI score (points)	1.16 (1.02–1.32)	**0.02**	**1.21 (1.05–1.39)**	**0.008**
CRP (mg/dl)	1.01 (0.99–1.031)	0.16	-	-
IL-6 (per 100 pg/ml)	1 (0.99–1.01)	0.59	-	-
PCT (ng/ml)	**1.03 (1.01–1.04)**	**0.003**	**1.02 (1.01–1.04)**	**0.004**
Mean arterial pressure (mmHg)	**0.98 (0.96–1.001)**	**0.06**	0.99 (0.97–1.01)	0.26
Heart rate (pulse/min)	**1.02 (1.001–1.04)**	**0.01**	**1.03 (1.01–1.04)**	**0.001**
Lactate (mmol/l)	**1.36 (1.04–1.77)**	**0.02**	1 (0.74–1.36)	0.99
Density_4−7 μm_ (10^− 2^ mm/mm^2^) ^#^	**0.98 (0.97–0.99)**	**< 0.001**	**0.98 (0.97–0.995)**	**0.006**
Density_4−7 μm_ dichotomized at median **	**4.96 (2.44–10.09)**	**< 0.001**	**4.80 (2.24–10.13)**	**< 0.001**
PBR_4−25 μm_ (per 0.1 μm) ^#^	**1.18 (1.03–1.34)**	**0.02**	1.13 (0.98–1.31)	0.1
PBR_4−25 μm_ dichotomized at median	1.39 (0.77–2.53)	0.28	-	-
MVHS (points)*	**0.57 (0.42–0.77)**	**< 0.001**	**0.6 (0.43–0.84)**	**0.003**

Bold indicates either variables selected for multivariable analysis (*p* < 0.10 in univariable analysis) or statistical significance in the final model (*p* < 0.05)

*To avoid multicollinearity, an alternate multivariable model was constructed in which the Microvascular Health Score (MVHS) replaced its individual components (capillary density and PBR), while all other covariates remained unchanged

** To avoid multicollinearity, an alternate multivariable model was constructed in which the dichotomized Density_4-7 μm_ variable replaced the continuous Density_4–7 μm_ variable. All other covariates remained unchanged

^#^ In a separate model including only the two microvascular variables (Density_4−7 μm_ and PBR_4−25 μm_), both remained independently associated with disease progression (Density_4−7 μm_: OR 0.98, 95% CI 0.97–0.99, *p* < 0.001; PBR_4−25 μm_ per 0.1 μm: OR 1.15, 95% CI 1.01–1.31, *p* = 0.04)

Abbreviations: CCI score = Charlson Comorbidity Index, CI = confidence interval, CRP = C-reactive protein, IL-6 = interleukin-6, MVHS = Microvascular Health Score, OR = odds ratio, PBR = perfused boundary region, PCT = procalcitonin

#### Subgroup analyses

Additional subgroup analyses were performed to explore potential differences related to the presence or absence of Sepsis-3 criteria at presentation. Therefore, we stratified the ED cohort into four groups according to clinical presentation (infection vs. sepsis) and subsequent disease progression during the first week (with vs. without progression) (Table 3).

**Table 3:** Emergency department (ED) patients presenting with suspected sepsis

Variable		ED infection patients			ED sepsis patients			
		W/o progression	With Progression	*p*-value	W/o progression	With progression	*p*-value	# *p*-value
Number of participants (n; %)		157	17	-	89	36	-	-
Female sex (n; %)		57 (36.3)	6 (35.3)	0.99	30 (33.7)	8 (22.2)	0.28	0.46
Age (years, median (IQR))		61 (44–73)	77 (57–83)	**0.01**	66 (55–79)	69 (56.5–79)	0.82	**0.03**
BMI (kg/m^2^, median (IQR))		25.8 (22.5–29.1)	23.5 (20.7–25.3)	0.11	25.9 (22.1–28.5)	24.45 (21.83–30.95)	0.78	0.43
CCI score (median (IQR))		0 (0–2)	1 (0.5–2.5)	**0.02**	1 (0–3.5)	1.5 (0–4.8)	0.56	**< 0.001**
qSOFA score (points, median (IQR))		0 (0–1)	0 (0–1)	0.44	0 (0–1)	1 (0–1)	0.08	**< 0.001**
SOFA score (points, median (IQR))		1 (0–2)	2 (1–4)	**0.007**	3 (2–5)	4 (2–8)	0.1	**< 0.001**
Length of hospital stay (days, median (IQR))		6 (4–10)	9.5 (5.3–18.3)	**0.03**	8 (5–13.8)	11 (7–24.8)	0.12	**< 0.001**
**Focus of infection (n; %)**								
Urinary tract		42 (26.8)	4 (23.5)	0.86	27 (30.3)	10 (27.8)	0.72	0.59
Respiratory tract		39 (24.8)	4 (23.5)		18 (20.2)	11 (30.6)		
Gastrointestinal tract		40 (25.5)	4 (23.5)		16 (18)	3 (8.33)		
Skin/soft tissue		10 (6.4)	1 (5.9)		8 (9)	4 (11.1)		
Other (unknown, CNS, cardiac, etc.)		26 (26.6)	4 (23.5)		20 (22.5)	8 (22.2)		
**Secondary outcomes**								
90-day mortality (n; %)		4 (2.5)	2 (11.8)	0.11	5 (5.6)	11 (30.6)	**< 0.001**	**< 0.001**
Inhospital mortality (n; %)		3 (2)	2 (11.8)	0.08	2 (2.2)	11 (30.6)	**< 0.001**	**< 0.001**
SOFA_max_ score (points, median (IQR))		-	3 (2–6)	-	-	6 (4–10)	-	-
ICU admission (n; %)		3 (1.9)	7 (41.2)	**< 0.001**	13 (14.6)	16 (44.4)	**< 0.001**	**< 0.001**
Composite outcome* (n; %)		3 (1.9)	17 (100)	**< 0.001**	2 (2.2)	36 (100)	**< 0.001**	**< 0.001**
**Laboratory data (median (IQR))**								
Creatinine (mg/dl)		1 (0.8–1.4)	1 (0.85–1.75)	0.50	1.5 (0.9–2.4)	1.85 (1.3–3.13)	0.11	**< 0.001**
CRP (mg/dl)		8.1 (3.1–18)	12.5 (8–16.2)	0.2	9.9 (3.9–17.1)	13.2 (7.2–29.5)	**0.03**	**0.03**
IL-6 (pg/ml)		53 (25.3–147.5)	141 (40.5–333.5)	0.07	117 (49–259.5)	181 (80–1779)	**0.02**	**< 0.001**
PCT (ng/ml)		0.23 (0.12–0.75)	0.29 (0.14–5.68)	0.15	1.04 (0.33–4.8)	6.37 (0.63–22.33)	**0.009**	**< 0.001**
Lactate (mmol/l)		1.1 (0.8–1.6)	1.5 (1.1–1.7)	0.053	1.3 (0.9–2.1)	1.4 (1.1–2.5)	0.14	**0.002**
**Macrocirculation data (median (IQR))**								
MAP (mmHg)		93.3 (84.7–101.7)	98.3 (81.8–105.2)	0.48	84.7 (73–99.5)	82.8 (63.8–92.8)	0.08	**< 0.001**
Heart Rate (pulse/min)		88 (77–100)	96 (77–113)	0.24	82 (74–96)	93 (83–111)	**0.007**	**0.02**
Respiratory Rate (breaths/min)		16 (14–18)	18 (15–24)	0.07	16 (14–20)	18 (15–21)	**0.04**	**0.02**
Temperature (°C)		37.6 (36.8–38.5)	37.5 (36.9–38.3)	0.72	37.4 (36.7–38.4)	38.1 (36.6–39.5)	0.25	0.64
**Microcirculation data (median (IQR))**								
Density_4−7 μm_ (10^− 2^ mm/mm^2^)		84.85 (63.61–117.39)	55.47 (33.50–66.82)	**< 0.001**	69.01 (50.32–87.18)	65.73 (51.75–77.63)	0.48	**< 0.001**
PBR_4−25 μm_ (µm)		2.30 (2.16–2.48)	2.32 (2.22–2.51)	0.54	2.37 (2.22–2.46)	2.41 (2.30–2.62)	0.052	**0.041**
MVHS (points)		2.43 (1.67–3.57)	1.17 (0.74–1.91)	**< 0.001**	2.05 (1.21–2.75)	1.62 (1.13–2.35)	0.14	**< 0.001**

Bold indicates statistical significance (*p* < 0.05)

# p value between all four groups (Kruskal-Wallis or chi square test)Abbreviations: BMI = body mass index, CCI score = Charlson Comorbidity Index, CRP = C-reactive protein, ED = emergency department, IL-6 = interleukin-6, IQR = interquartile range, MAP = mean arterial pressure, MVHS = microvascular health score, PBR = perfused boundary region, PCT = procalcitonin, qSOFA score = quick SOFA score, SOFA score = Sequential Organ Failure Assessment score

**Fig. 2:**
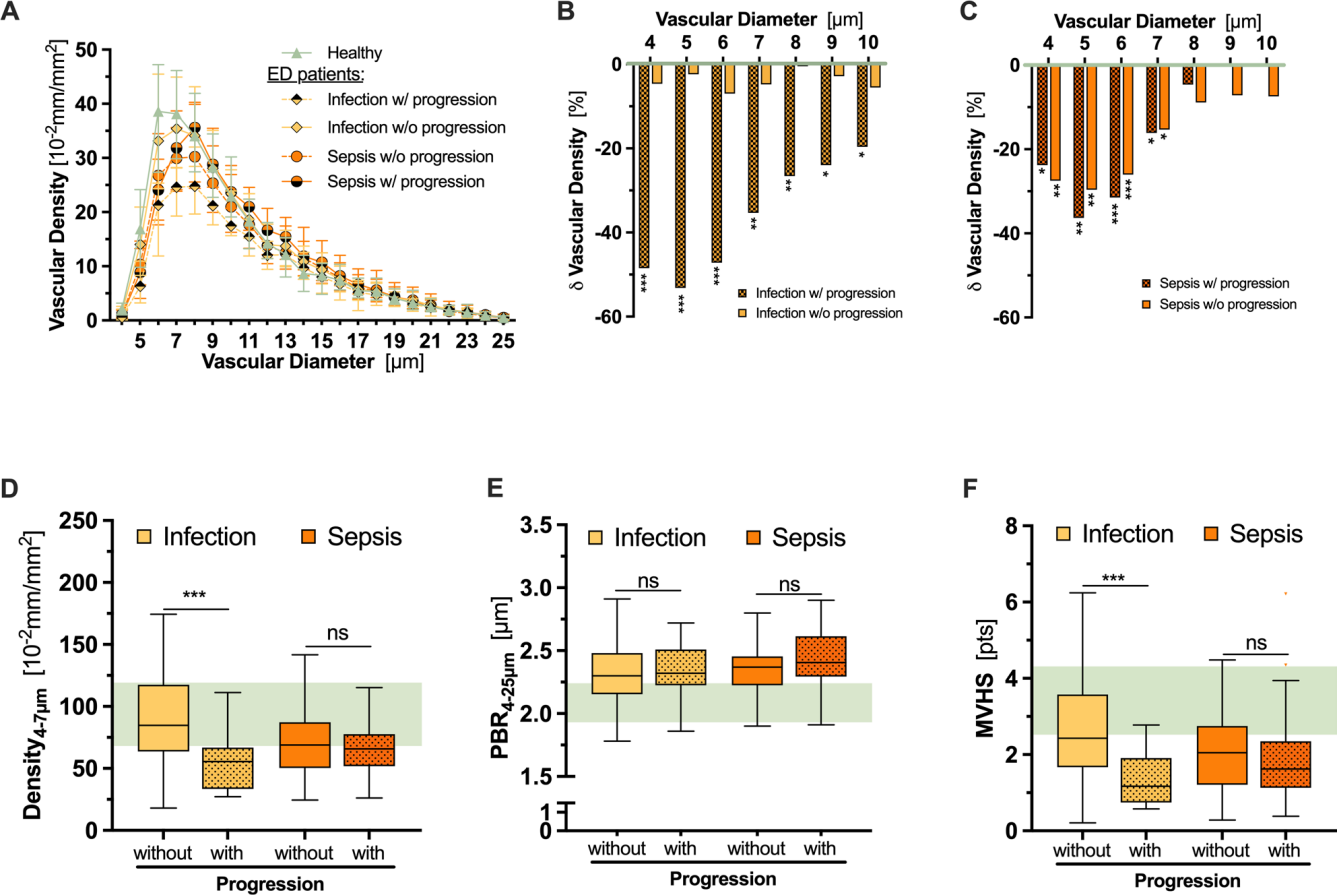
Microvascular pattern of ED patients with or without disease progression stratified by infection severity (infection or sepsis) at presentation. (**A**) Median [IQR] vascular density of the diameter classes from 4 to 25 μm in healthy controls (green; *n* = 50) and ED patients presenting with infection without sepsis (yellow; *n* = 174) or sepsis (orange; *n* = 125). Half-shaded symbols indicate patients with disease progression (infection: *n* = 17/174; sepsis: *n* = 36/125). (**B**-**C**) Bar charts showing the percentage reduction of vascular density of the diameter classes from 4 to 10 μm in ED patients with (dotted) and without disease progression presenting with (**B**) infection (yellow) or (**C**) sepsis (orange) compared to healthy controls (green). Boxplots showing (**D**) capillary density (Density_4−7 μm_), (**E**) perfused boundary region (PBR_4−25 μm_), and (**F**) Microvascular Health score (MVHS) in ED patients with infection (yellow) or sepsis (orange) with (dotted) or without disease progression. The IQR of healthy controls is highlighted in green. * *p* < 0.05, ** *p* < 0.01, *** *p* < 0.001, ns: not significant

Capillary density was mostly preserved in patients with infection without progression, whereas all other groups (infection with progression, sepsis with and without progression) showed markedly reduced values (Table 3, Fig. 2). In contrast, PBR_4−25 μm_ showed a small, but significant increase across all four groups (Table 3). As a composite metric, the observed differences in MVHS appeared largely influenced by changes in capillary density.

In the infection subgroups, univariable and multivariable logistic regression analyses confirmed that capillary density (OR 0.96 [0.93–0.98], *p* < 0.001) and MVHS (OR 0.31 [0.15–0.62], *p* < 0.001) both predicted progression into sepsis (Supp. Table 2). The MVHS (AUC 0.79, *p* < 0.0001) outperformed traditional biomarkers such as procalcitonin (AUC 0.61, *p* = 0.16) and interleukin-6 (AUC 0.64, *p* = 0.06).

### Secondary outcomes

#### Mortality

**Fig. 3:**
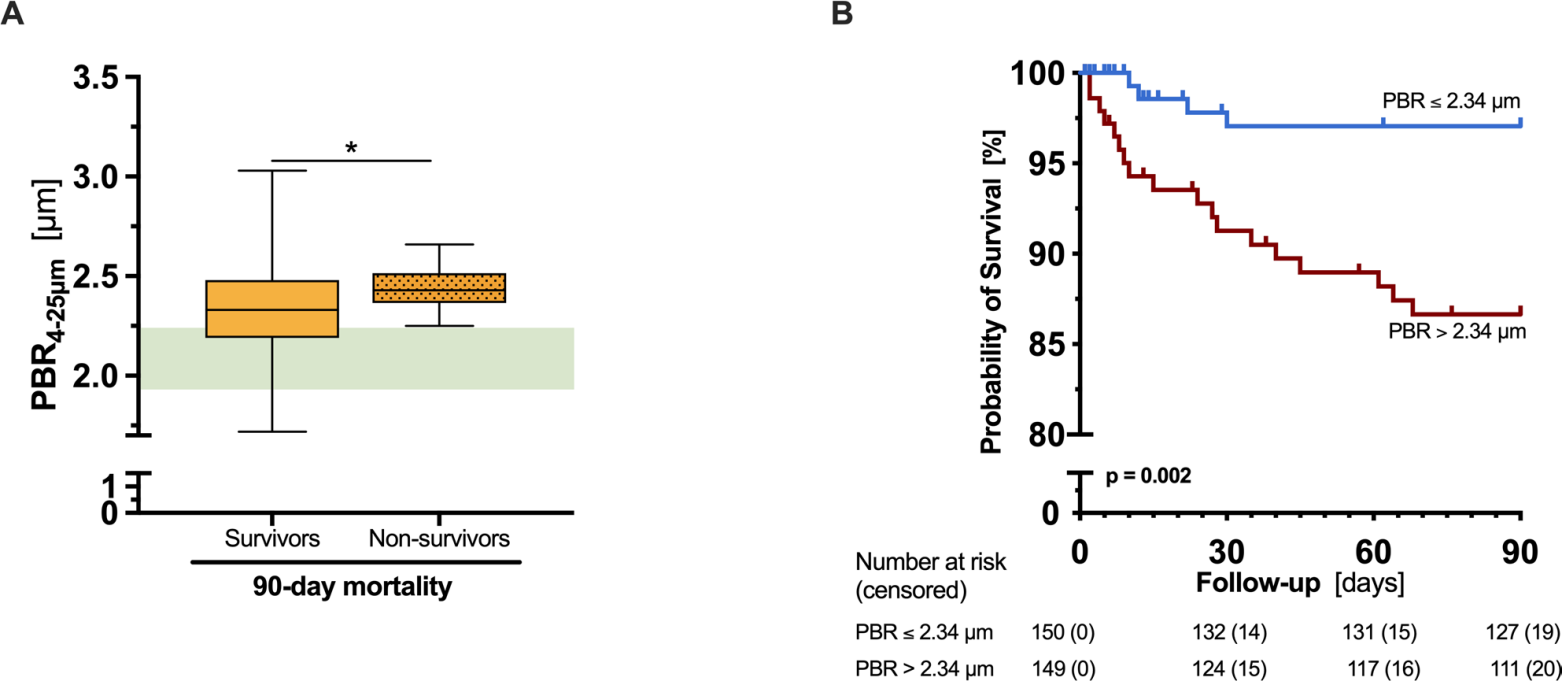
90-day mortality according to endothelial glycocalyx dimensions on admission. (**A**) Boxplot showing PBR_4−25 μm_ values of survivors and non-survivors. (**B**) Kaplan-Meier curve showing 90-day mortality of ED patients stratified by PBR_4−25 μm_ values (less versus greater than the median [2.34 μm]). Survival curves were compared using the log-rank test. *p < 0.05

PBR_4–25 μm_ dichotomized at the median was independently associated with both 90-day mortality (OR 6.42, [1.71–24.1], *p* = 0.006) and in-hospital mortality (OR 5.2, [1.4–19.31], *p* = 0.01) in logistic regression analyses (Supp. Tables 3 and 4). Capillary density showed no prognostic value, whereas analysis of PBR_4−25 μm_ on a continuous scale showed a non-significant trend. Consistently, non-survivors had significantly higher PBR_4−25 μm_ values than survivors (90-day: 2.43 [2.37–2.51] vs. 2.33 [2.19–2.48] µm, *p* = 0.04; in-hospital: 2.43 [2.37–2.53] vs. 2.34 [2.19–2.48] µm, *p* = 0.04) (Fig. 3, Supp. Figure 5). Figure 3 and Supp. Figure 5 illustrate Kaplan-Meier survival curves for 90-day and in-hospital mortality, respectively, stratified by PBR (less versus higher than median [2.34 μm]).

#### ICU admission

In the ED cohort, higher lactate, heart rate, and lower mean arterial pressure, were independently associated with ICU admission during hospital stay, while IL-6 and PCT were associated in univariable analysis only. A trend for the MVHS seen in the univariable analysis did not remain significant in the multivariable model (Supp. Table 5).

#### Composite outcome of disease progression or in-hospital mortality

We next explored the relationship between microvascular alterations and a composite outcome of disease progression within the first week or in-hospital mortality (n = 58/299; 19.4%). Patients were stratified according to the cohort median of capillary density (Density_4–7 μm_: 75.08 10⁻² mm/mm²) and glycocalyx thickness (PBR_4–25 μm_: 2.34 μm), resulting in four distinct microvascular phenotypes (Table 4). Patients with intact microvasculature (low PBR_4−25 μm_ *and* high Density_4−7 μm_) at the time of ED presentation had the lowest incidence (4/76 patients, 5.3%) and a markedly reduced likelihood of the composite outcome (OR 0.17 [95% CI: 0.07–0.4], *p* < 0.001). In contrast, patients with combined glycocalyx damage *and* reduced capillary density showed the highest event rate (23/75 patients; 30.7%) and a significantly increased likelihood of the composite outcome (OR = 2.39, 95% CI: 1.30–4.42, *p* = 0.007). Isolated abnormalities of either capillary density (20/74 patients; 27%) or glycocalyx thickness (11/74 patients; 14.9%) were associated with weaker and non-significant risk estimates.

Diagnostic performance characteristics of the four phenotypes are summarized in Table 4. Notably, the intact microvasculature phenotype identified a subgroup of ED patients with particularly low observed rate of early clinical deterioration.

**Table 4:** Microvascular phenotypes and association with the composite outcome

Microvascular phenotype *	Event Rate *n*/*N* (%)	Odds Ratio (95% CI)	*p* value	Sensitivity (%)	Specificity (%)	PPV (%)	NPV (%)
Intact microvasculature	4/76 (5.3%)	0.17 (0.07–0.47)	0.0002	93.1	29.9	24.2	94.7
Isolated impaired glycocalyx	11/74 (14.9%)	1.51 (0.75–3.13)	0.31	19	73.9	14.9	79.1
Isolated reduced capillary density	20/74 (27%)	1.82 (0.95–3.41)	0.06	34.5	77.6	27	83.1
Reduced capillary density & impaired glycocalyx	23/75 (30.7%)	2.39 (1.30–4.42)	0.007	39.7	78.4	30.7	84.4

* Diagnostic performance metrics were calculated considering the presence of each phenotype as the test condition and the composite outcome as the endpoint. For the protective phenotype (intact microvasculature), the absence of this phenotype was considered the positive test condition in the diagnostic analysis. Odds ratios were calculated for each phenotype compared with the remainder of the ED cohort. Abbreviations: CI = confidence interval, NPV = negative predictive value, PPV = positive predictive value

## Discussion

This prospective observational study demonstrates that quantitative sublingual videomicroscopy identifies clinically relevant microvascular alterations at the time of ED presentation in patients with suspected infection or sepsis. Despite low baseline SOFA scores and largely preserved macrocirculatory parameters, patients already exhibited significant impairment of capillary density and glycocalyx integrity compared with healthy and disease controls. Microvascular variables remained independently associated with disease progression despite adjustment for macrocirculatory parameters, consistent with the concept of loss of hemodynamic coherence, in which restoration of systemic hemodynamics does not necessarily reflect normalization of microvascular perfusion [19]. Importantly, these alterations were not merely epiphenomena of advanced critical illness but carried prognostic information, particularly in patients presenting with infection who subsequently deteriorated during the first week of hospitalization.

By applying a class-wise, diameter-resolved analysis and focusing on the smallest perfused capillaries (4–7 μm), our approach enabled the detection of subtle yet prognostically meaningful microvascular abnormalities at an early clinical stage. This quantitative strategy extends prior work that predominantly relied on semi-quantitative visual scoring systems and focused on ICU populations with established sepsis or multi-organ failure [6–8, 12, 20]. In contrast, our findings suggest that relevant microvascular injury is already present at first hospital contact and may precede clinically overt organ dysfunction as captured by conventional scores.

From a pathophysiological perspective, the sublingual microvasculature may act as a sensitive integrator of the host response to infection. Microvascular dysfunction represents one of the earliest system-wide manifestations of inflammatory, immunologic, and circulatory stress [21, 22]. Endothelial activation, glycocalyx degradation, and capillary flow heterogeneity are therefore expected to emerge before sustained macrocirculatory failure or overt organ dysfunction becomes clinically apparent. The early detectability of these alterations in our cohort supports the concept that microvascular injury marks a critical transition phase in the evolution from localized infection toward systemic disease.

A key finding of this study is the stage-dependent prognostic performance of quantitative microvascular assessment. Microvascular variables, including capillary density, PBR, and the composite MVHS, showed robust associations with disease progression in patients presenting with infection, whereas their discriminative value was markedly attenuated in patients who already fulfilled sepsis criteria at ED admission. Importantly, this observation should be interpreted in the context of current Sepsis-3 definitions. In our cohort, the distinction between infection and sepsis at presentation was frequently driven by minimal differences in SOFA scores, often reflecting the absence or presence of a single organ dysfunction point. Such dichotomization may insufficiently capture the continuous and dynamic nature of early disease biology. Against this background, the strong prognostic discrimination observed prior to overt sepsis may indicate that quantitative microvascular alterations reflect a more granular and biologically relevant measure of early disease severity than categorical sepsis classifications [23]. In established sepsis, more diffuse and advanced microvascular injury [24] may lead to homogenization of abnormalities and compression of the dynamic range, thereby limiting prognostic discrimination. Alternative explanations, including ceiling effects or confounding by early therapeutic interventions, cannot be fully excluded but are consistent with this interpretation.

Distinct microvascular phenotypes were associated with divergent clinical trajectories. Notably, our data indicate that alterations in glycocalyx integrity and capillary density do not invariably co-occur. A substantial proportion of patients exhibited discordant profiles – either increased PBR with preserved capillary density or reduced capillary density despite intact glycocalyx measurements – and these isolated abnormalities were not independently associated with the worst outcomes. Consistent with this observation, both capillary density and PBR remained independently associated with disease progression in regression analyses, indicating that glycocalyx alterations provide complementary prognostic information beyond capillary density alone. Together, these findings support the concept that glycocalyx integrity and capillary density reflect partially independent dimensions of microvascular injury. Importantly, this interpretation is consistent with our previous work demonstrating that alterations in glycocalyx-related parameters and microvascular perfusion metrics associate with distinct biological signatures and do not necessarily evolve in parallel, even within comparable clinical phenotypes [9, 20, 25–29]. In contrast, patients with concurrent impairment of both parameters – indicating combined disruption across these domains – had a markedly increased risk of disease progression and mortality. When both regulatory axes are compromised simultaneously, compensatory capacity may be exceeded, providing a plausible explanation for the synergistic increase in clinical risk observed in this subgroup.

From a clinical perspective, quantitative microvascular assessment may serve as an adjunctive screening and risk stratification tool in patients presenting to the ED with suspected infection, particularly in those with equivocal clinical findings or borderline risk profiles. Rather than replacing established clinical assessment or decision pathways, microvascular assessment could add physiologically meaningful information in patients who do not clearly meet criteria for either low-risk management or immediate intensive care. An intact microvascular phenotype may support standard ward management or de-escalation of monitoring intensity, whereas early identification of combined glycocalyx damage and capillary rarefaction could justify closer surveillance, earlier ICU referral, or inclusion in trials targeting endothelial protection. Conceptually, this resembles a rule-out/rule-in paradigm analogous to D-dimer testing [30], where preserved microvascular integrity identifies patients at very low risk of early deterioration, thereby supporting more efficient allocation of critical care resources. Importantly, similar to D-dimer concentrations, quantitative microvascular parameters may reflect continuous biological risk rather than adherence to arbitrarily defined diagnostic thresholds, identifying clinically relevant vascular injury before patients meet categorical criteria for sepsis.

## Limitations

This study has several limitations. First, it was conducted at a single tertiary center, which may limit generalizability. In addition, enrollment was non-consecutive and dependent on operator availability, such that selection bias cannot be excluded. However, results were consistent and robust in multivariable analyses. Second, although we adjusted for relevant clinical covariates, residual confounding, particularly related to pre-existing endothelial vulnerability in conditions such as diabetes or vascular disease, cannot be excluded. Moreover, although the cohort size was substantial, the number of events for some exploratory subgroup analyses was limited and the analyses might have been underpowered. Third, videomicroscopy is technically demanding and susceptible to artefacts related to motion or pressure. However, standardized acquisition protocols, operator training, and automated quality control were employed to mitigate these issues. Fourth, while serial measurements were performed in exploratory analyses, temporal trajectories and dynamic changes in MVHS did not provide additional prognostic information beyond the baseline assessment, limiting conclusions regarding treatment responsiveness or microvascular recovery. Although MVHS has previously shown good discriminatory performance [12], differences in the present cohort appeared to be largely driven by capillary density, and superiority of the composite score over the individual microvascular parameters could not be consistently demonstrated. Larger studies are needed to confirm its incremental prognostic value. Finally, as an observational study, causality between microvascular injury and subsequent organ dysfunction cannot be inferred and requires confirmation in interventional studies.

## Conclusion and outlook

In summary, quantitative sublingual microvascular alterations are detectable at ED presentation in patients with infection or sepsis and identify individuals at increased risk of early clinical deterioration, particularly during the transition from infection to sepsis. These findings suggest that microvascular injury represents an early and clinically relevant manifestation of endothelial stress that is incompletely captured by conventional scores and biomarkers. Incorporation of quantitative microvascular parameters such as PBR, capillary density, or the composite MVHS into early clinical workflows may improve risk stratification and enable biological enrichment for future trials targeting endothelial protection or microvascular repair. Multicenter validation and interventional studies are warranted to determine whether early detection of microvascular changes and subsequent correction of microvascular dysfunction can ultimately modify clinical outcomes. 

## Supplementary Information

Supplementary Material 1

## Data Availability

The datasets used and/or analyses during the current study are available from the corresponding author on reasonable request.

## Abbreviations

AUC: Area under the curve
BMI: Body mass index
CBV: Capillary blood volume
CCI score: Charlson Comorbidity Index
CI: Confidence interval
CoV: Coefficient of variation
CRP: C-reactive protein
D: Diameter
ED: Emergency department
EDGE: Early Detection of Glycocalyx and microvascular damage in suspected sepsis in the Emergency department
EGC: Endothelial glycocalyx.
FDR: False discovery rate
Hb: Haemoglobin
ICU: Intensive care unit
IL-6: Interleukin-6
IQR: Interquartile range
MAP: Mean arterial pressure
MVHS: Microvascular health score
NPV: Negative predictive value
OR: Odds ratio
PBR: Perfused boundary region
PCT: Procalcitonin
PPV: Positive predictive value
qSOFA score: Quick Sequential Organ Failure Assessment score
RBC: Red blood cell
ROC: Receiver operating characteristic
SDF: Sidestream dark field
SOFA score: Sequential Organ Failure Assessment score
SSC: Surviving sepsis campaign
