# Supplementary material for: Early detection of glycocalyx and microvascular damage in suspected sepsis in the emergency department: the EDGE study
Source: Crit Care. 2026 Apr 17;30:241. doi: 10.1186/s13054-026-05989-9 (PMC13154576; doi:10.1186/s13054-026-05989-9)
Supplement: Supplementary file 1 — Supplementary Material 1 [file 13054_2026_5989_MOESM1_ESM.docx]

Supplemental materials

**Early Detection of Glycocalyx and microvascular damage in suspected sepsis in the Emergency department: the EDGE study**

Melina Mascha Scarbeck^1*^, Marc-David Künnemann^1,2*^, Anna M. Hunkemöller^1, 3^, Carolin Christina Drost^1^, Alexander Lukasz^1^, Marcel Birkner^1^, Manfred Fobker^4^, Jerzy-Roch Nofer^4, 5^, Hans Vink^6^, Hermann Pavenstädt^1^, Philipp Kümpers^1#^, Alexandros Rovas^1#^

^1^Department of Medicine D, Division of General Internal and Emergency Medicine, Nephrology, and Rheumatology, University Hospital Münster, Albert-Schweitzer-Campus 1, 48149, Münster, Germany

^2^Clinic of Radiology, University Hospital Münster, Albert-Schweitzer-Campus 1, 48149, Münster, Germany.

^3^Institute of Diagnostic and Interventional Radiology, Hannover Medical School, 30625 Hannover, Germany.

^4^Central Laboratory Facility, University Hospital Münster, Albert-Schweitzer-Campus 1, 48149, Münster, Germany

^5^Institute for Laboratory Medicine, Marien-Hospital, Niels-Stensen-Kliniken, Osnabrück, Germany

^6^Glycocalyx Research Institute, Alpine, USA

**Supp. Methods**

*Capillary recruitment and dynamic MVHS*

In healthy individuals, the velocity of RBCs (V_RBC_) in capillaries (Density_4-7 µm_) remains relatively constant, irrespective of the associated V_RBC_ in feed vessels (D ≥ 10 µm). Conversely, the V_RBC_ in capillaries of patients with sepsis is directly proportional to the V_RBC_ in feeding vessels, suggesting that the number of perfused capillaries in sepsis is *fixed* and is unaffected by local variations in tissue metabolic demand. The calculation of CR (in %) is thus based on the following formula: 1 − slope(V_RBC_ (Density_4-7 µm_), V_RBC_ (D ≥ 10 µm) [1]. For the second phase of the study, the scope of the software was expanded, enabling the capture of sufficient low- and high-flow situations per subject to generate intra-individual regression slopes and the calculation of CR on a per-patient basis. The dynamic version of the capillary blood volume (CBV_dynamic_) is obtained by multiplying the CBV x (1 + CR). The quotient of the CBV_dynamic_ divided by the PBR_dynamic_ (a flow-corrected version of the PBR [1]) results in the MVHS_dynamic_.

**Supp. Table 1:** Primary outcome of ED patients in the pooled ED infection/sepsis cohort

| **Variable** | **ED infection/sepsis** | **Primary Outcome** | | ***p-value** |
| --- | --- | --- | --- | --- |
|  |  | **W/o progression** | **With progression** |  |
| Number of participants (n; %) | 299 | 246 (82.3) | 53 (17.7) | - |
| Female sex (n; %) | 101 (33.8) | 87 (35.4) | 14 (26.4) | 0.27 |
| Age (years, median (IQR)) | 64  (51-77) | 63 (48-76) | 70 (57-80.5) | **0.04** |
| BMI (kg/m^2^, median (IQR)) | 25.5  (22.2-29) | 25.8  (22.4-29.1) | 23.9 (21.9-27.7) | 0.18 |
| CCI score (median (IQR)) | 1 (0-3) | 0 (0-2) | 1 (0-4) | **0.01** |
| qSOFA score (points, median (IQR)) | 0 (0-1) | 0 (0-1) | 1 (0-1) | **0.005** |
| SOFA score (points, median (IQR)) | 2 (1-3) | 2 (1-3) | 3 (2-5) | **<0.001** |
| Length of hospital stay (days, median (IQR)) | 8 (5-13) | 7 (4-11) | 10 (6.8-22) | **0.001** |
| Sepsis on admission (n; %) | 125 (41.8) | 89 (36.2) | 36 (67.9) | **<0.001** |
| **Focus of infection** (n; %) | | | | |
| Urinary tract | 83 (27.8) | 69 (28) | 14 (26.4) | 0.78 |
| Respiratory tract | 72 (24.1) | 57 (23.2) | 15 (28.3) |  |
| Gastrointestinal tract | 63 (21.1) | 56 (22.8) | 7 (13.2) |  |
| Skin/soft tissue | 24 (8) | 19 (7.7) | 5 (9.4) |  |
| Other (unknown, CNS, cardiac, etc.) | 57 (19.1) | 45 (18.3) | 12 (22.6) |  |
| **Secondary Outcomes** | | | | |
| 90-day mortality (n; %) | 22 (7.4) | 9 (3.7) | 13 (24.5) | **<0.001** |
| In-hospital mortality (n; %) | 18 (6) | 5 (2) | 13 (24.5) | **<0.001** |
| SOFA_max_ score (points, median (IQR)) | 2 (1-4) | - | 5 (3-9) | **-** |
| ICU admission (n; %) | 39 (13.04) | 16 (6.5) | 23 (43.4) | **<0.001** |
| Composite outcome^+^ (n; %) | 58 (19.4) | 5 (2.03) | 53 (100) | **<0.001** |
| **Laboratory data (median (IQR))** | | | | |
| Creatinine (mg/dl) | 1.2 (0.9-1.8) | 1.2 (0.8-1.7) | 1.6 (1-2.5) | **0.003** |
| CRP (mg/dl) | 9.6 (4-18.5) | 9.2 (3.6-17.6) | 12.7 (7.6-22.1) | **0.004** |
| IL-6 (pg/ml) | 90 (39-226) | 72 (36-203) | 159 (70-907) | **<0.001** |
| PCT (ng/ml) | 0.44 (0.16-2.33) | 0.4 (0.14-1.59) | 2.31 (0.30-14.97) | **<0.001** |
| Lactate (mmol/l) | 1.25 (0.9-1.7) | 1.2 (0.9-1.7) | 1.5 (1.1-2.1) | **0.002** |
| **Macrocirculation data (median (IQR))** | | | | |
| MAP (mmHg) | 91.3 (79.83-100) | 92.33  (80.42-100) | 88 (69.5-99.17) | 0.05 |
| Heart Rate (pulse/min) | 88 (77-102) | 86 (75-100) | 96 (82-111) | **0.009** |
| Respiratory Rate (breaths/min) | 16 (15-20) | 16 (14-18) | 18 (16-21) | **0.002** |
| Temperature (°C) | 37.5 (36.7-38.5) | 37.5  (36.7-38.5) | 37.6 (36.7-38.9) | 0.49 |
| **Microvascular data (median (IQR))** | | | | |
| Density_4-7 µm_ (10^-2^ mm/mm^2^) | 75.08  (55.65-100.30) | 77.9  (59.52-105) | 62.2  (43.01-74.55) | **<0.001** |
| PBR_4-25 µm_ (µm) | 2.34  (2.2-2.49) | 2.34  (2.18-2.48) | 2.39 (2.26-2.58) | **0.03** |
| MVHS (points) | 2.14  (1.39-3.16) | 2.24  (1.53-3.38) | 1.52 (0.99-2.19) | **<0.001** |

*p values were calculated between the two ED groups (without and with progression)

^+^composite outcome: disease progression or in-hospital mortality

Abbreviations: BMI = body mass index, CCI score = Charlson Comorbidity Index, CNS = central nervous system, CRP = C-reactive protein, ED = emergency department, ICU = intensive care unit, IL-6 = interleukin-6, IQR = interquartile range, qSOFA = quick Sequential Organ Failure Assessment Score, MVHS = microvascular health score, PBR = perfused boundary region, PCT = procalcitonin, SOFA = Sequential Organ Failure Assessment Score

**Supp. Table 2:** Univariate and multivariate logistic regression of the infection cohort (n=174), with progression into sepsis as the dependent variable

|  | **Univariable** | | **Multivariable** | |
| --- | --- | --- | --- | --- |
| **Independent variables** | **OR (95% CI)** | **p-value** | **OR (95% CI)** | **p-value** |
| Sex (female/male) | 0.96 (0.34-2.73) | 0.93 | - | - |
| Age (years) | **1.03 (1-1.07)** | **0.05** | 1.03 (0.99-1.07) | 0.1 |
| CCI score (points) | 1.21 (0.95-1.55) | 0.12 | - | - |
| CRP (mg/dl) | 1.02 (0.96-1.08) | 0.53 | - | - |
| IL6 (per 100 pg/ml) | 0.99 (0.95-1.04) | 0.78 | - | - |
| PCT (ng/ml) | **1.07 (1-1.15)** | **0.04** | 1.08 (1-1.17) | 0.05 |
| Mean arterial pressure (mmHg) | 1.02 (0.98-1.1) | 0.46 | - | - |
| Heart rate (pulse/min) | 1.02 (0.99-1.05) | 0.13 | - | - |
| Lactate (mmol/l) | 1.5 (0.85-2.67) | 0.16 | - | - |
| Density_4-7 µm_ (10^-2^ mm/mm^2^) | **0.96 (0.94-0.98)** | **<0.001** | **0.96 (0.93-0.98)** | **<0.001** |
| Density_4-7 µm_ dichotomized at median ** | **29.67**  **(3.83-229.74)** | **0.001** | **41.79**  **(3.66-477.08)** | **0.003** |
| PBR_4-25 µm_ (per 0.1 µm) | 1.05 (0.83-1.32) | 0.68 | - | - |
| PBR_4-25 µm_ dichotomized at median | 0.87 (0.32-2.40) | 0.79 | - | - |
| MVHS (points)* | **0.32 (0.17-0.62)** | **<0.001** | **0.31 (0.15-0.62)** | **<0.001** |

*In an alternate multivariable model, the Microvascular Health Score (MVHS) was entered instead of capillary density to avoid multicollinearity. All other covariates remained unchanged.

** In an alternate multivariable model, the dichotomized version was entered instead of Density_4-7 µm_ (10^-2^ mm/mm^2^) to avoid multicollinearity. All other covariates remained unchanged.

Abbreviations: CCI score = Charlson Comorbidity Index, CRP = C-reactive protein, IL-6 = interleukin-6, MVHS = Microvascular Health Score, PBR = perfused boundary region, PCT = procalcitonin.

**Supp. Table 3:** Univariable and multivariable logistic regression with 90-day mortality in the ED cohort (n=299) as the dependent variable

|  | **Univariable** | | **Multivariable** | |
| --- | --- | --- | --- | --- |
| **Independent variables** | **OR (95% CI)** | **p-value** | **OR (95% CI)** | **p-value** |
| Sex (female/male) | 0.91 (0.36-2.31) | 0.84 | - | - |
| Age (years) | **1.05 (1.02-1.09)** | **0.003** | **1.06 (1.02-1.1)** | **0.04** |
| CCI score (points) | **1.21 (1.02-1.43)** | **0.03** | 0.98 (0.79-1.21) | 0.83 |
| CRP (mg/dl) | 1.01 (0.99-1.03) | 0.26 | - | - |
| IL6 (per 100 pg/ml) | 1 (1-1.01) | 0.37 | - | - |
| PCT (ng/ml) | **1.02 (1-1.03)** | **0.09** | 1.01 (0.98-1.03) | 0.72 |
| Mean arterial pressure (mmHg) | **0.96 (0.93-0.99)** | **0.003** | **0.97 (0.94-0.996)** | **0.03** |
| Heart rate (pulse/min) | 1 (0.98-1.03) | 0.61 | - | - |
| Lactate (mmol/l) | **1.58 (1.15-2.18)** | **0.01** | **1.47 (1.0-2.15)** | **0.048** |
| Density_4-7 µm_ (10^-2^ mm/mm^2^) | 1 (0.99-1.01) | 0.8 | - | - |
| Density_4-7 µm_ dichotomized at median | 1.50 (0.62-3.62) | 0.37 | - | - |
| PBR_4-25 µm_ (per 0.1 µm) | 1.12 (0.97-1.41) | 0.11 | - | - |
| PBR_4-25 µm_ dichotomized at median | **5.02 (1.66-15.20)** | **0.004** | **6.42 (1.71-24.1)** | **0.006** |
| MVHS (points) | 0.89 (0.62-1.27) | 0.51 | - | - |

Abbreviations: CCI score = Charlson Comorbidity Index, CRP = C-reactive protein, IL-6 = interleukin-6, MVHS = Microvascular Health Score, PBR = perfused boundary region, PCT = procalcitonin.

**Supp. Table 4:** Univariable and multivariable logistic regression with in-hospital mortality in the ED cohort (n=299) as the dependent variable

|  | **Univariable** | | **Multivariable** | |
| --- | --- | --- | --- | --- |
| **Independent variables** | **OR (95% CI)** | **p-value** | **OR (95% CI)** | **p-value** |
| Sex (female/male) | 0.97 (0.35-2.68) | 0.96 | - | - |
| Age (years) | **1.05 (1.01-1.09)** | **0.009** | **1.05 (1.01-1.09)** | **0.01** |
| CCI score (points) | 1.12 (0.93-1.36) | 0.24 | **-** | **-** |
| CRP (mg/dl) | 1.01 (0.99-1.03) | 0.34 | - | - |
| IL6 (per 100 pg/ml) | 1 (1-1-01) | 0.44 | - | - |
| PCT (ng/ml) | **1.02 (1-1.04)** | **0.045** | 1.01 (0.99-1.03) | 0.25 |
| Mean arterial pressure (mmHg) | **0.96 (0.94-0.99)** | **0.02** | 0.97 (0.94-1.0) | 0.08 |
| Heart rate (pulse/min) | 1.01 (0.99-1.04) | 0.29 | - | - |
| Lactate (mmol/l) | **1.57 (1.12-2.18)** | **0.008** | 1.45 (1-2.12) | 0.052 |
| Density_4-7 µm_ (10^-2^ mm/mm^2^) | 1.04 (0.99-1.02) | 0.52 | **-** | - |
| Density_4-7 µm_ dichotomized at median | 1 (0.39-2.59) | 1 | **-** | - |
| PBR_4-25 µm_ (per 0.1 µm) | **1.20 (0.97-1.5)** | **0.09** | 1.26 (0.98-1.61) | 0.08 |
| PBR_4-25 µm_ dichotomized at median * | **5.45 (1.54-19.24)** | **0.008** | **5.2 (1.4-19.31)** | **0.01** |
| MVHS (points) | 1.05 (0.74-1.50) | 0.79 | **-** | - |

Abbreviations: CCI score = Charlson Comorbidity Index, CRP = C-reactive protein, IL-6 = interleukin-6, MVHS = Microvascular Health Score, PBR = perfused boundary region, PCT = procalcitonin.

* In an alternate multivariable model, the dichotomized version was entered instead of PBR_4-25 µm_ (per 0.1 µm) to avoid multicollinearity. All other covariates remained unchanged.

**Supp. Table 5:** Univariate and multivariate logistic regression with ICU admission in the ED cohort (n=299) as the dependent variable

|  | **Univariable** | | **Multivariable** | |
| --- | --- | --- | --- | --- |
| **Independent variables** | **OR (95% CI)** | **p-value** | **OR (95% CI)** | **p-value** |
| Sex (female/male) | 1.62 (0.82-3.21) | 0.17 | - | - |
| Age (years) | 1.01 (0.99-1.03) | 0.2 | - | - |
| CCI score (points) | 1.09 (0.94-1.26) | 0.27 | - | - |
| CRP (mg/dl) | 1.01 (0.99-1.02) | 0.36 | - | - |
| IL6 (per 100 pg/ml) | **1.01 (1-1.02)** | **0.01** | 1 (1-1.03) | 0.3 |
| PCT (ng/ml) | **1.02 (1.01-1.04)** | **0.01** | 1.02 (1-1.04) | 0.08 |
| Mean arterial pressure (mmHg) | **0.97 (0.95-0.99)** | **0.008** | **0.97 (0.95-1)** | **0.007** |
| Heart rate (pulse/min) | **1.03 (1.01-1.05)** | **0.001** | **1.02 (1-1.04)** | **0.02** |
| Lactate (mmol/l) | **2.02 (1.47-2.77)** | **<0.001** | **1.94 (1.41-2.66)** | **<0.001** |
| Density_4-7 µm_ (10^-2^ mm/mm^2^) | 0.99 (0.98-1) | 0.12 | **-** | **-** |
| Density_4-7 µm_ dichotomized at median | 1.73 (0.87-3.44) | 0.12 | **-** | **-** |
| PBR_4-25 µm_ (per 0.1 µm) | 0.85 (0.19-3.74) | 0.83 | - | - |
| PBR_4-25 µm_ dichotomized at median * | 0.95 (0.49-1.86) | 0.88 | - | - |
| MVHS (points) | **0.74 (0.55-1.01)** | **0.056** | 0.86 (0.62-1.18) | 0.34 |

Abbreviations: CCI score = Charlson Comorbidity Index, CRP = C-reactive protein, IL-6 = interleukin-6, MVHS = Microvascular Health Score, PBR = perfused boundary region, PCT = procalcitonin.

**Supp. Figures**


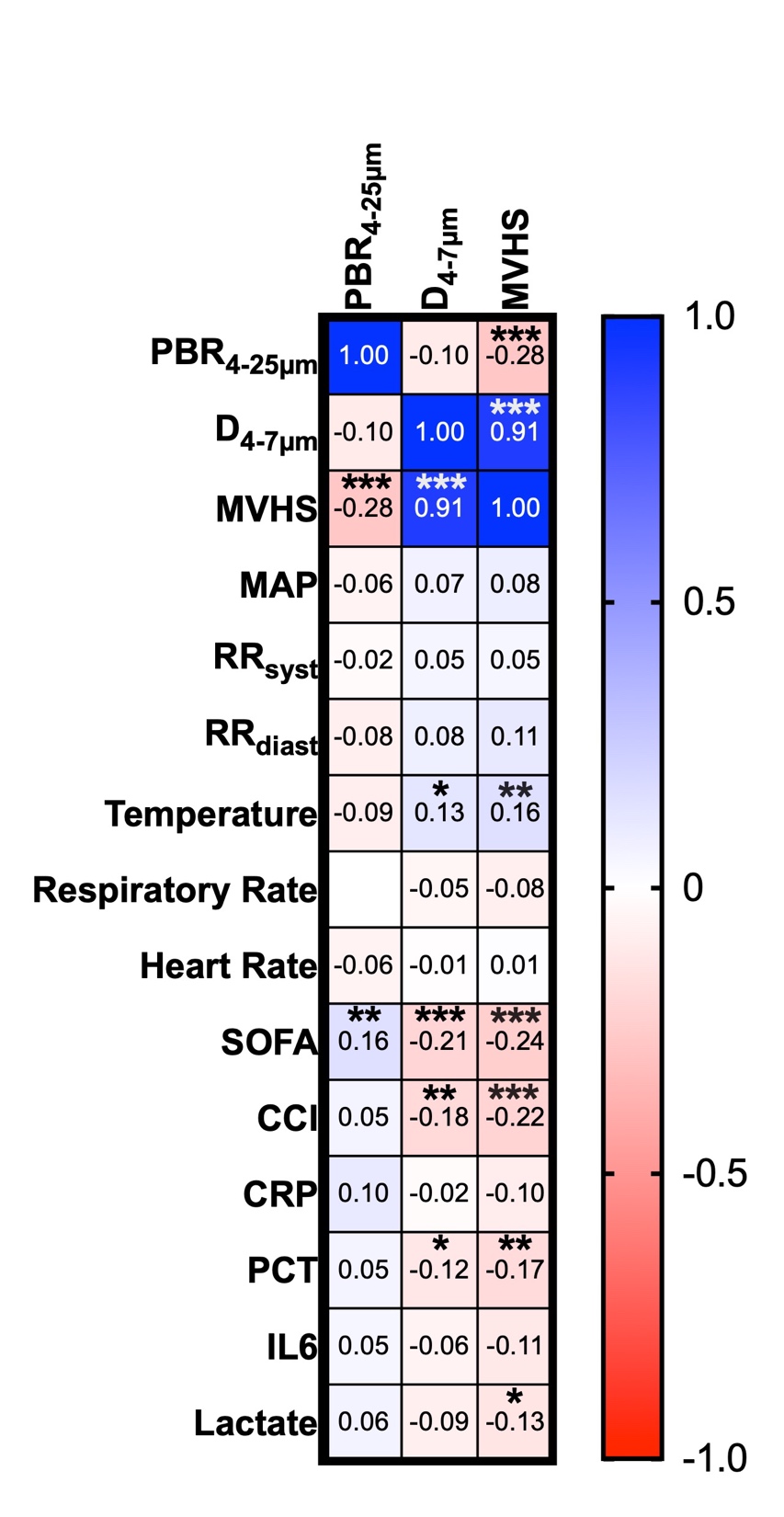


**Supp. Fig. 1:** Correlation matrix (Spearman test) showing comparison of microvascular variables with macrovascular, laboratory and clinical parameters in the ED cohort (n = 299). Statistical significance is marked as following: * *p* < 0.05, ** *p* < 0.01, *** *p* < 0.001


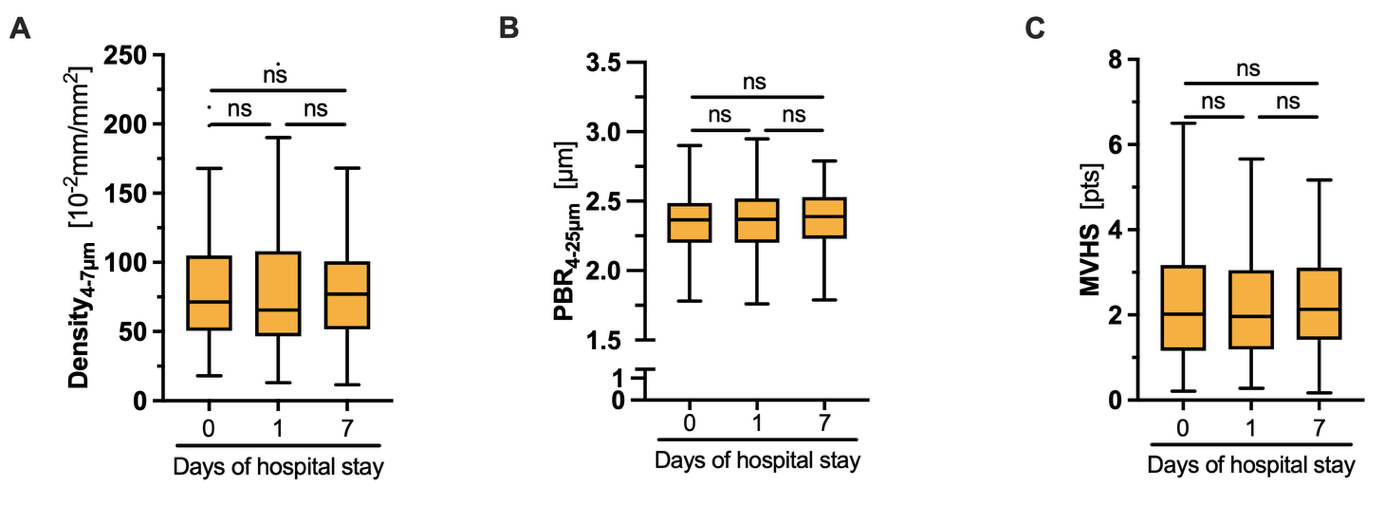


**Supp. Fig. 2: Trajectory of microvascular variables during the first week of the hospital stay.** (A-C) Boxplots of microvascular variables at presentation and days 1 (n = 136) and 7 (n = 67). ns: not significant

**
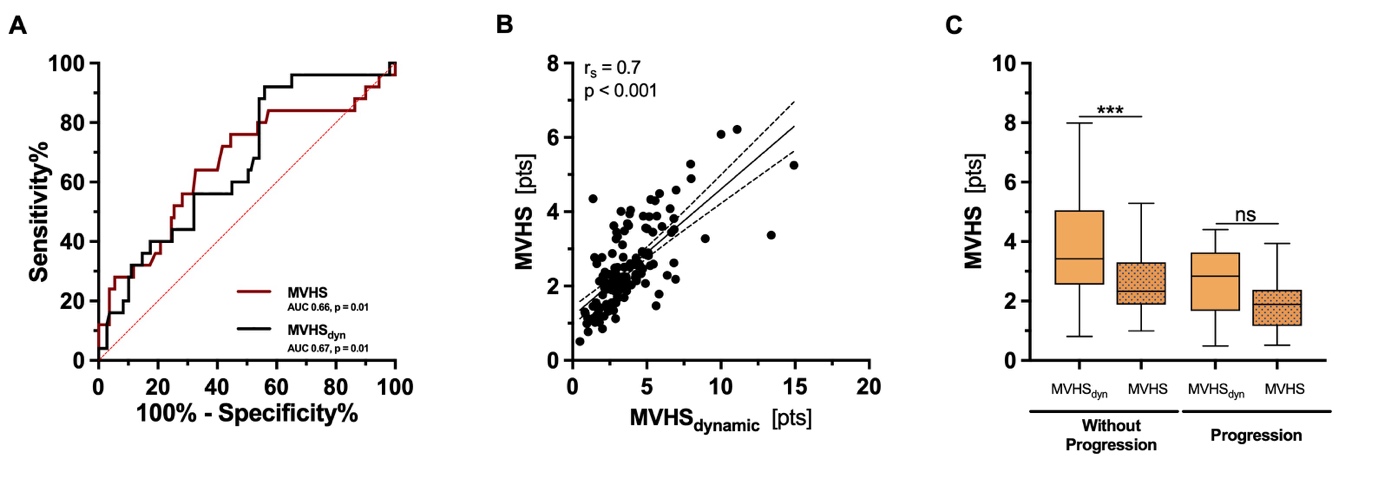
**

**Supp. Fig. 3: MVHS and MVHS_dynamic_ regarding disease progression in the subgroup of the ED cohort (n = 134).** (A) Receiver operating characteristic (ROC) curves showing the discriminative performance of MVHS and MVHS_dynamic_ for predicting disease progression. (B) Correlation of MVHS and MVHS_dynamic_ in the ED cohort. (C) Boxplots of MVHS_dynamic_ and MVHS stratified by ED patients with or without disease progression. **** p < 0.001*


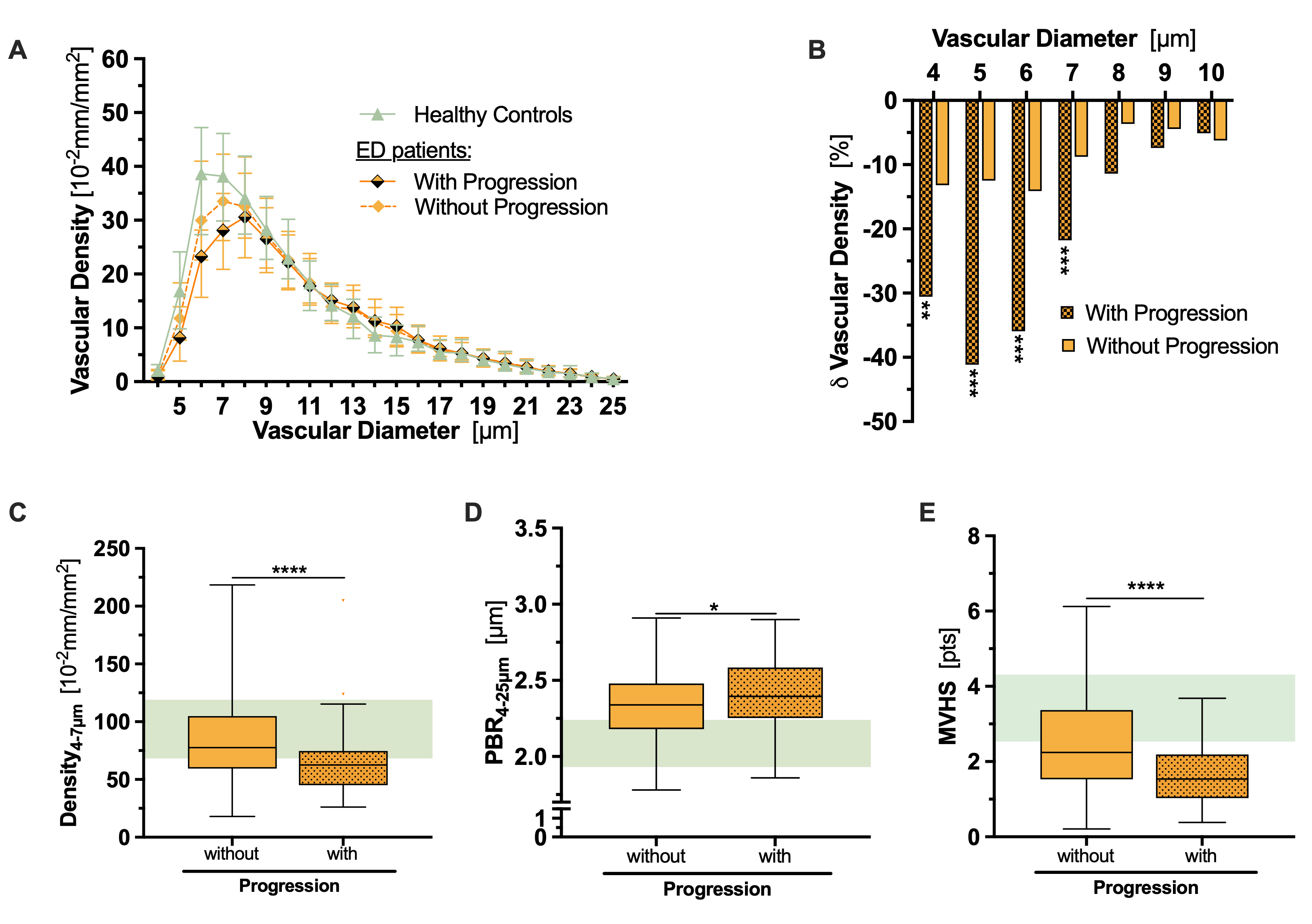


**Supp. Fig. 4 Microvascular pattern of ED patients with / without disease progression**

(A) Median [IQR] vascular density of the diameter classes from 4 to 25 µm in healthy controls (green; n = 50) and ED patients with (brown; n = 53) and without (orange; n = 246) disease progression. (B) Bar charts showing the percentage reduction of vascular density of the diameter classes from 4 to 10µm in ED patients with (brown) and without (orange) disease progression compared to healthy controls. Boxplots showing (C) capillary density (Density_4-7µm_), (D) perfused boundary region (PBR_4-25µm_), and (E) Microvascular Health score (MVHS) in ED patients with (brown; n = 53) and without (orange; n = 246) disease progression. The IQR of healthy controls is highlighted in green. ** p < 0.05, ** p < 0.01, *** p < 0.001*


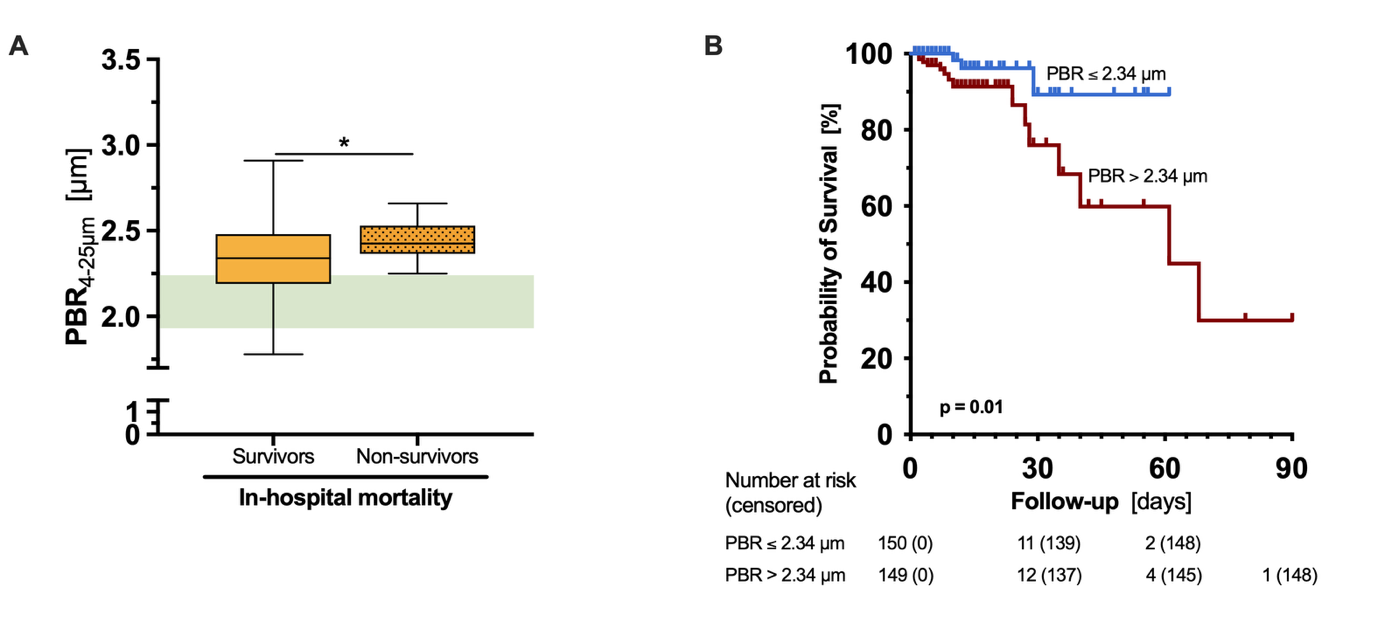


**Supp. Fig. 5: In-hospital mortality according to endothelial glycocalyx dimensions on admission**

(A) Boxplot showing PBR_4-25µm_ values of survivors and non-survivors. (B) Kaplan-Meier curve showing in-hospital mortality of ED patients stratified by PBR_4-25µm_ values (less versus greater than the median [2.34 µm]). Survival curve was compared using the log-rank test. ** p < 0.05*

1. Rovas A, Sackarnd J, Rossaint J, Kampmeier S, Pavenstadt H, Vink H, Kumpers P: **Identification of novel sublingual parameters to analyze and diagnose microvascular dysfunction in sepsis: the NOSTRADAMUS study**. *Crit Care* 2021, **25**(1):112.
